# Local Recurrence of Breast Cancer 52 Years after Halsted Mastectomy: Is There a Role for More Aggressive Ipsilateral Surveillance?

**DOI:** 10.1155/2011/107370

**Published:** 2011-11-13

**Authors:** Shailesh Agarwal, Edward W. Nelson, Jayant P. Agarwal

**Affiliations:** ^1^Pritzker School of Medicine, University of Chicago, Chicago, IL, USA; ^2^Division of General Surgery, University of Utah, Salt Lake City, UT, USA; ^3^Division of Plastic and Reconstructive Surgery, University of Utah, 30 North 1900 East 3B400, Salt Lake City, UT 84132, USA

## Abstract

We present the longest reported case of breast cancer recurrence, 52 years after initial diagnosis, in a patient initially treated with Halsted mastectomy. Observation and palpation of the chest wall resulted in late presentation, and this patient went on to demonstrate metastatic disease. Current surveillance guidelines lack specific recommendations regarding monitoring of the ipsilateral chest wall. In addition, the growing utilization of breast reconstruction poses an additional challenge to surveillance strategies of the ipsilateral breast. However, the emergence of MRI may present a new opportunity to identify ipsilateral recurrence. The changing landscape of breast cancer therapy warrants guidance from groups of national import such as ASCO, in the surveillance of breast cancer patients.

## 1. Introduction

Breast cancer recurrence surveillance in mastectomy patients remains an open issue in the literature. Local recurrence rates in patients treated with mastectomy are reported to be from 2.3 to as high as 30 percent [1–9]. While the American Society of Clinical Oncology (ASCO) recommends regular mammograms for all women previously treated with breast-conserving therapy [10, 11], these guidelines are not specific to surveillance of the ipsilateral chest wall in the mastectomy patient. Chest wall recurrence in these patients may only be detected via observation or palpation, and the chest wall cannot be adequately imaged using current mammography techniques. 

The wide variety of available techniques for breast cancer therapy has given physicians greater flexibility to treat breast cancer, both oncologically and aesthetically with breast reconstruction. However, this has also presented greater challenges to physicians to develop treatment-specific protocols for recurrence surveillance. 

The following case highlights a local/regional recurrence 52 years after initial Halsted mastectomy. This is the longest reported case of local recurrence in the indexed literature to our knowledge—two other publications report recurrences after 48 and 32 years [12, 13]. Additionally, this case raises several key points which deserve attention in the further development of postmastectomy surveillance recommendations.

## 2. Methods

### 2.1. Case

**Figure 1:**
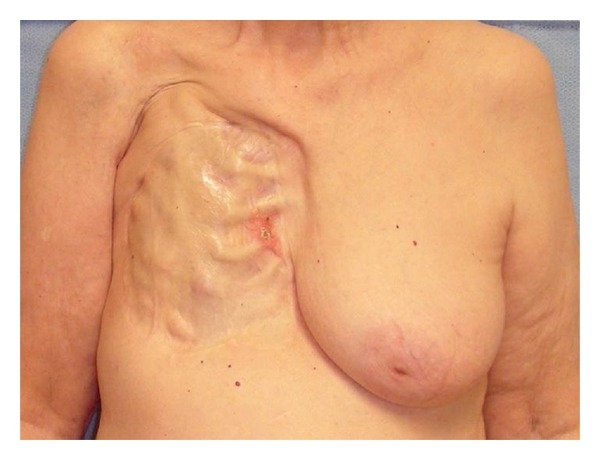
An 80-year-old woman with local chest wall recurrence of breast cancer 52 years after Halsted mastectomy.

An 80-year-old woman with a previous history of breast cancer presented with an erythematous, indurated lesion on the right chest wall. She had previously undergone a radical mastectomy on the right breast 52 years earlier for breast carcinoma; additionally she was treated with radiation therapy at that time. The patient had undergone only placement of a split-thickness skin graft placed directly over her ribs as reconstruction after her initial resection. The patient has undergone routine mammographic examination of her contralateral breast, but no further imaging was performed on her ipsilateral mastectomy site since the time of her initial treatment. The patient presented to a dermatologist in August of 2007 after development of a 3 cm × 3 cm red, scaly lesion on the right chest wall at the medial aspect of the skin graft (Figure 1). The patient noted that this lesion had become painful and friable with intermittent bleeding.

**Figure 2:**
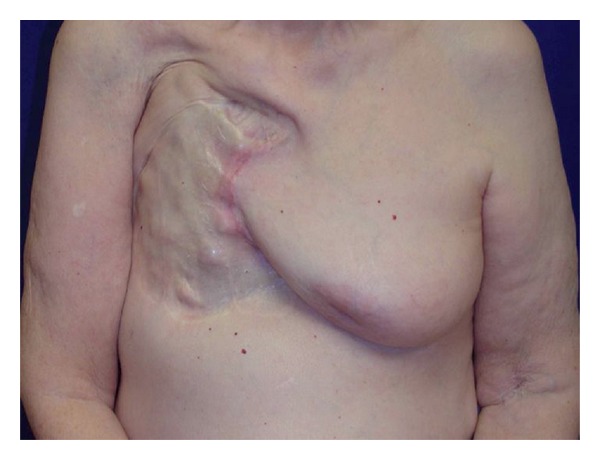
The same patient after chest wall lesion excision and local tissue rearrangement using the skin from her contralateral breast.

A shave biopsy of the skin graft was performed which revealed an invasive, moderately differentiated squamous cell carcinoma. Wide local excision of the erythematous lesion, including the underlying rib, was then performed. This specimen revealed a poorly differentiated adenocarcinoma consistent with locally recurrent breast cancer. The deep margin was positive, and the tumor was found by stains to be ER+/PR+ and Her2/neu+. A CT scan further revealed the presence of pulmonary metastases. The patient underwent local tissue rearrangement using the contralateral breast skin to close the defect, followed by chemotherapy for treatment of her pulmonary metastases (Figure 2).

## 3. Discussion

In the case presented here, local recurrence occurred 52 years after mastectomy for breast cancer treatment. The risk of locoregional recurrence in breast cancer patients following mastectomy has been reported to be between 2.3 and 30 percent [1–9]. Some studies report that up to 80 percent of patients treated with newer skin-sparing mastectomy techniques have some remaining breast tissue [14, 15]. However, no standard practice exists for surveillance of the ipsilateral chest wall. 

Based on data from the Breast Cancer Treatment Effectiveness in Older Women (BOW) study, Field et al. have shown that, by the fourth year after treatment, only 60% of breast cancer survivors aged 65 and over visit an oncologist or breast surgeon [16]. Less than 70% of women receive a surveillance mammogram during their fourth year of followup after breast cancer treatment [16]. Nearly a one-third reduction in breast cancer mortality rate has been shown to be associated with each additional surveillance mammogram for breast cancer survivors over 65 years [17]. Data from the Surveillance, Epidemiology, and End Results (SEER) database demonstrate a decreased risk of breast-cancer-specific mortality in women who receive a mammogram within a one- or two-year interval before death [18]. It should be noted that these studies address the use of surveillance mammography and are not specific to ipsilateral surveillance. In mastectomy patients, the lack of available tissue obviates the use of mammography for ipsilateral surveillance. In the case reported here, observation and palpation were inadequate for recurrence detection. By the time the lesion was evident on clinical exam, the underlying ribs were involved and pulmonary metastasis had occurred. This raises an important question regarding the appropriate method of ipsilateral chest wall surveillance. While observation and palpation may eventually detect recurrence, it may not be sufficient for timely detection.

Given the increased rates of postmastectomy breast reconstruction, we must also consider surveillance of the reconstructed breast cancer survivor. The literature shows that between 8 and 16 percent of breast cancer patients treated with mastectomy undergo postmastectomy reconstruction, and this number continues to increase [19–24]. Currently, there are no specific guidelines for postreconstructive surveillance of the ipsilateral breast [5, 10, 11]. A recent systematic review concluded that there is currently little evidence studying surveillance in the reconstructed breast and that further research is needed [25]. Furthermore, reconstruction itself can be performed using a variety of techniques. Implant-based reconstruction can be performed using saline or silicone implants which in addition may be placed either deep or superficial to the pectoralis muscle. Autologous flap reconstruction should be performed using a variety of the patient's own tissues including abdomen (transverse abdominus rectus myocutaneous flap), buttocks (gluteal myocutaneous flap), and even thigh (gracilis flap). These variables confound the reliability of the manual exam and mammogram. 

Finally, magnetic resonance imaging (MRI) offers a newer modality for breast cancer screening in certain indicated patient subgroups; however, current guidelines from ASCO recommend against the use of MRI for general breast cancer surveillance [10]. The utility of MRI for surveillance in the reconstructed breast still remains unaddressed by current ASCO guidelines.

## 4. Conclusion

Surveillance for local breast cancer recurrence remains a debated topic in the scientific literature. We report a case of local recurrence in an 80-year-old woman, 52 years after initial treatment with Halsted mastectomy; observation and palpation of the chest wall resulted in late presentation, and this patient went on to demonstrate metastatic disease. Current surveillance guidelines lack specific recommendations regarding monitoring of the ipsilateral chest wall. In addition, the growing utilization of breast reconstruction poses an additional challenge to surveillance strategies of the ipsilateral breast. However, the emergence of MRI may present a new opportunity to identify ipsilateral recurrence. The changing landscape of breast cancer therapy warrants guidance from groups of national import such as ASCO, in the surveillance of breast cancer patients.

##  Disclosure

None of the authors have financial interests to disclose, and there were no external sources of funding provided for this project. No products or devices are mentioned in this paper. No animals were used in this study, and no ethics approval was required.
